# Preeclampsia-Associated Multivessel Spontaneous Coronary Artery Dissection

**DOI:** 10.1177/2324709619874624

**Published:** 2019-09-11

**Authors:** Rajeev Virender Seecheran, Jessica Kawall, Divya Ramadhin, Valmiki Krishna Seecheran, Sangeeta Anjali Persad, Sasha Savitri Lalla, Naveen Anand Seecheran

**Affiliations:** 1 North Central Regional Health Authority, Mt. Hope, Trinidad and Tobago; 2 Advanced Cardiovascular Institute, Port of Spain, Trinidad and Tobago; 3 University of the West Indies, St. Augustine, Trinidad and Tobago

**Keywords:** pregnancy-associated spontaneous coronary artery dissection, PASCAD, spontaneous coronary artery dissection, SCAD, ST-segment elevation myocardial infarction, STEMI, multivessel dissection, preeclampsia

## Abstract

Pregnancy-associated spontaneous coronary artery dissection (PASCAD) accounts for less than 5% of spontaneous coronary artery dissection cases and is comparatively more fulminant or clinically aggressive. Several factors associated with PASCAD include black ethnicity, multiparity, hypertension, advanced maternal age, and age at first childbirth. This atypical case highlights a preeclamptic patient presenting with an ST-segment elevation myocardial infarction in which multivessel dissection of both the left anterior descending and right coronary arteries were deemed co-culprit lesions for the index event.

## Introduction

Spontaneous coronary artery dissection (SCAD) is rapidly gaining traction as an emerging but often neglected cause of myocardial infarction, especially in young women.^1^ It has a complex pathophysiology, variable prognosis, and distinct treatment strategy as compared with typical acute coronary syndromes.^2-4^ It is defined as a spontaneous dissection of the coronary artery wall with an accumulation of blood within the false lumen, which can compress the true lumen to varying degrees.^1^ Of further concern is pregnancy-associated SCAD (PASCAD), which accounts for less than 5% of cases and is comparatively more fulminant or clinically aggressive.^5^

SCAD was previously, incorrectly, believed to be very rare and almost exclusively associated with pregnancy. Furthermore, intracoronary imaging sparingly aided diagnosis. Therefore, the preceding reports of SCAD prevalence on coronary angiography of 0.2% to 1.1% vastly underestimated its true prevalence with the PASCAD type affecting almost 2 per 100 000 pregnancies.^6-8^

This atypical case highlights a preeclamptic patient presenting with an ST-segment elevation myocardial infarction in which multivessel dissection of both the left anterior descending (LAD) artery and right coronary artery (RCA) were deemed co-culprit lesions for the index event.

## Case Report

A 31-year-old multiparous Caribbean black woman with 32-week gestational age presented to the emergency department with abrupt-onset, severe angina and dyspnea. Her vital signs reflected a hypertensive emergency with systolic blood pressures of approximately 180 mm Hg, tachycardia of 126 beats per minute, and tachypnea of 24 breaths per minute with oxygen saturation of 98% on room air. On physical examination, her abdomen was consistent with that of a third trimester gestation while the gynecologic (pelvic) examination was deferred until the Women’s Health team arrived. There was no aortic regurgitation murmur, differential blood pressures among the extremities, with a mildly elevated jugular venous pressure of 9 cm, crackles extending to the mid-bilateral lung zones, and pretibial edema. Her brief review of systems was unremarkable. A portable chest radiograph revealed florid pulmonary edema, while urine proteinuria was significantly positive.

**Figure 1.**
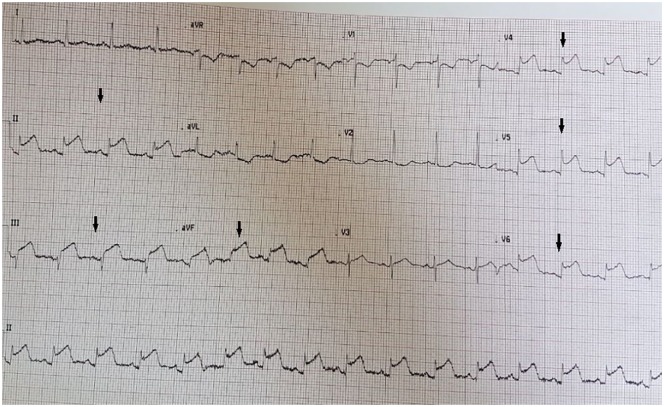
Index presentation electrocardiogram (ECG). There is marked 5- to 6-mm convex ST-segment elevation in the inferior leads of II, II and aVF (left-sided arrows) with ST depression in leads V1-V2, suggestive of an inferoposterior myocardial infarction. Additionally, there is similar ST-segment elevation in leads V4-V6 (right-sided arrows box), suggestive of an anterolateral myocardial infarction. Overall, the ECG displays a diffusely ischemic picture typically reflected in left main or multivessel coronary artery disease.

**Figure 2.**
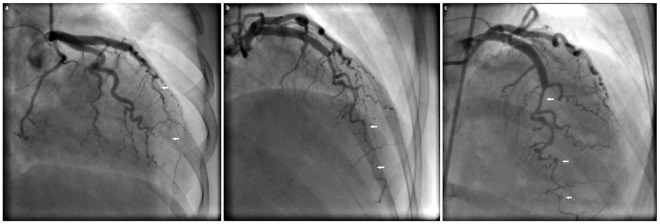
Cineangiography series of the left coronary artery (LCA). (a) Right anterior oblique (RAO) angiographic view of the left anterior descending (LAD) artery with the white arrows subtending the type 2A pregnancy-associated spontaneous coronary artery dissection (PASCAD). (b) “Steep” RAO angiographic view of the LAD artery with the white arrows subtending the type 2A PASCAD. (c) Straight cranial view of the LAD artery with the white arrows subtending the type 2A PASCAD.

**Figure 3.**
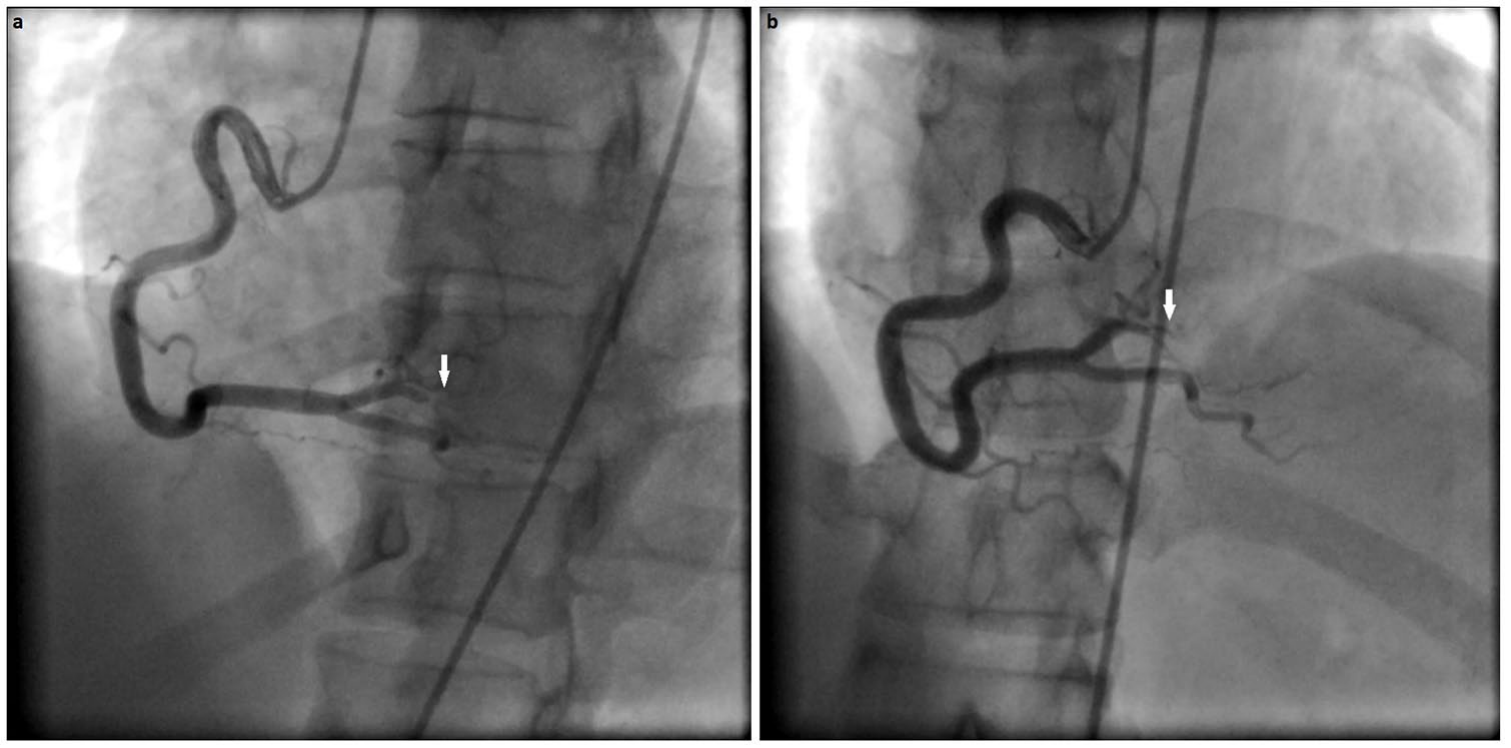
Cineangiography series of the right coronary artery (RCA). (a) Left anterior oblique angiographic view of the RCA with the white arrow indicating the type 2A pregnancy-associated spontaneous coronary artery dissection (PASCAD). (b) Right anterior oblique angiographic view of the RCA artery with the white arrow indicating the type 2A PASCAD.

An emergent 12-lead electrocardiogram indicated a sinus tachycardia with marked 5- to 6-mm, convex ST-segment elevation in both the inferior (II, III, and aVF) and anterolateral (V4-V6) leads (see Figure 1). An urgent bedside transthoracic echocardiogram revealed mild to moderate hypokinesis of the respective inferior and anterior territories with an estimated ejection fraction of 40% to 45%. She proceeded immediately to the cardiac catheterization laboratory (Artis zee, Siemens AG Healthineers, Munich, Germany) as per the institution’s primary percutaneous coronary intervention (PCI) protocol for coronary angiography. In the interim, guideline-directed medical therapy was initiated with a titratable, intravenous nitroglycerin and low-dose labetalol infusion after which her angina subsided, and dyspnea waned after achieving near-normotensive control. No thrombolytic agents were administered. Angiography revealed a diffuse, mid to transapical LAD artery type II A SCAD pattern (see Figure 2) as well as a proximal posterior left ventricular branch of the RCA type II A SCAD pattern (see Figure 3). There was no intravascular ultrasonography, optical coherence tomography, or cardiothoracic surgical backup capabilities on site.

The tentative diagnosis was dual culprit SCADs of the LAD and RCA, attributed to preeclampsia with a resultant type 2 myocardial infarction. Our multidisciplinary team opted for a noninvasive, conservative medical management strategy that included an antithrombotic regimen of aspirin, clopidogrel, and enoxaparin with β-blockade. Angiotensin-converting enzyme inhibition, mineralocorticoid receptor antagonists, and high-intensity statin therapy were avoided in light of anticipated delivery within 1 month and subsequent breastfeeding.

During her 10-day hospitalization, the patient underwent extensive investigations that included secondary hypertension and vasculitides workup, all of which were all normal. Both human immunodeficiency testing and toxicology screen for drugs of abuse were negative. A predischarge transthoracic echocardiogram displayed normalization of ejection fraction from 60% to 65% amid steadily declining cardiac biomarkers. Her angina and dyspnea resolved over the ensuing course, and she was subsequently discharged on guideline-directed medical therapy with an early outpatient clinic appointment to both cardiology and the high-risk obstetrical unit. She successfully underwent vaginal delivery 6 weeks later without any maternal or fetal complications and was extensively counseled against further pregnancy with which she was in consensus.

## Discussion

It is challenging to ascertain the bona fide impact of PASCAD due to its unknown prevalence, coupled with inadequate intracoronary imaging techniques for accurate diagnosis; thus, the preceding reports of PASCAD prevalence on coronary angiography likely underestimate this condition.^6,7^ A recent editorial by Saw suggests that PASCAD accounts for less than 5% of cases in modern, contemporary series.^5^ PASCAD can occur from any time during the antepartum period extending up to 2 years postpartum.^3,9,10^

The pathophysiology of PASCAD is both complex and multifactorial. The mechanistic effects involve both pregnancy-related hemodynamics coupled with a neurohormonal milieu, which compromises the coronary vascular integrity. Progesterone attenuates the arterial media by inhibiting collagen synthesis and thus affecting the elastic fibrin content and ratio whereas estrogen augments both the prothrombotic state and matrix metalloproteinase concentration.^11,12^ Acute straining during labor and delivery akin to the Valsalva maneuver can accentuate impulse and shear stress. It is imperative to recognize that these alterations may persist for 6 months despite delivery before returning to a pregestational state and, possibly, result in cumulative exposures in subsequent pregnancies with an elevated risk.^13^

The average age of presentation with an index event in a patient with PASCAD is approximately 33 years, which is typically younger than the SCAD cohort, the substantial majority of which occurred in the postpartum period.^14,15^ Several factors associated with PASCAD include black ethnicity, multiparity, hypertension, advanced maternal age, and age at first childbirth.^8^ The clinical spectrum of PASCAD ranges from subtle to fulminant and comprises angina, acute coronary syndromes, ventricular arrhythmias, chronic heart failure, cardiogenic shock, and sudden cardiac death.^9^

In several comprehensive series, 50% to 75% of patients presented with ST-segment myocardial infarctions, in-hospital mortality ranged from 4% to 50%, multivessel involvement occurred in 35% to 70%, left main involvement in 5% to 46%, ventricular fibrillation in 16%, and cardiogenic shock in 24% to 33%, which frequently required mechanical circulatory support in 28%. Twenty-eight percent of the patients presented with sudden cardiac death. Patients generally had a mean left ventricular ejection fraction of 40%.^13,15-18^ PCI was not widely performed, with suboptimal success rates, approaching 50%. Coronary artery bypass grafting is also not common (37%). Most patients were treated conservatively; however, a large subgroup ultimately required revascularization. Only 65% of patients had angiographic healing, and persistent SCAD often appeared in those with recurrent ischemic symptoms. Fetal mortality was significant at 11% with neonatal complications in nearly one third.^18,19^

There are several compelling characteristics that differentiate PASCAD from its non–pregnancy-related counterpart. First, patients with PASCAD are, on average, a decade younger, and its incidence is a fraction of the SCAD cohort (~5%). Also, there is a predilection for left main, multivessel, and proximal vessel involvement. These acute presentations tend to be more sinister, owing to the greater, jeopardized territory at risk. Procedural success rates for PCI are similar between the two, with surveillance angiography revealing lower healing rates.^20^ Our patient’s obstetrical diagnosis was consistent with severe preeclampsia as per the American College of Obstetrics and Gynecologists (ACOG) criteria given her hypertension, proteinuria, and florid pulmonary edema, which subsequently resolved.^21-24^ She also possessed several risk factors for PASCAD, including ethnicity, multiparity, and gestational hypertension, which evolved to preeclampsia. In addition, the rationale for deferring PCI incorporated the patient’s abating symptomatology and improved hemodynamics combined with the lack of advanced imaging modalities (intravascular ultrasonography and optical coherence tomography) and on-call cardiothoracic surgical team in a limited-resource setting in Trinidad. Both culprit lesions were classified as type II A as per the angiographic schema, illustrated by Saw et al.^1,25^ As aforementioned, the patient was treated with a conservative, noninvasive strategy with a negative workup for secondary hypertension and vasculitides. Her cardiovascular regimen included anti-anginals of long-acting nitrates and carvedilol in addition to the antithrombotic pharmacotherapy, which entailed aspirin and clopidogrel. Therapeutic enoxaparin was administered throughout her hospitalization and discontinued on discharge.^26,27^ Other agents such as prasugrel, ticagrelor, and rivaroxaban were considered; however, they were decided against given their uncertain track record in PASCAD and breastfeeding.^23,26,28^

Differentiating between PASCAD and its non-pregnancy type is critical because patients with PASCAD are markedly at higher risk of major adverse cardiovascular events and may require emergent invasive treatment strategies.

## Conclusion

In summary, PASCAD is an exceedingly rare complication and represents a considerably high-risk subgroup. The pathophysiology is not fully elucidated; however, it is generally associated with devastating maternal and fetal complications, often requiring emergent revascularization and mechanical circulatory support. Further research is required on this enigmatic and potentially catastrophic condition to ascertain optimal treatment strategies.

## References

[1] Saw J Mancini GBJ Humphries KH. Contemporary review on spontaneous coronary artery dissection. J Am Coll Cardiol. 2016;68:297-312. doi:10.1016/j.jacc.2016.05.034 27417009

[2] Tweet MS Gulati R Hayes SN. What clinicians should know αbout spontaneous coronary artery dissection. Mayo Clin Proc. 2015;90:1125-1130. doi:10.1016/j.mayocp.2015.05.010 26250728

[3] Saw J Aymong E Sedlak T, et al Spontaneous coronary artery dissection: association with predisposing arteriopathies and precipitating stressors and cardiovascular outcomes. Circ Cardiovasc Interv. 2014;7:645-655. doi:10.1161/CIRCINTERVENTIONS.114.001760 25294399

[4] Thygesen K Alpert JS Jaffe AS, et al Fourth universal definition of myocardial infarction (2018). Circulation. 2018;138:e618e651. doi:10.1161/CIR.0000000000000617 30571511

[5] Saw J. Pregnancy-associated spontaneous coronary artery dissection represents an exceptionally high-risk spontaneous coronary artery dissection cohort. Circ Cardiovasc Interv. 2017;10:e005119. doi:10.1161/CIRCINTERVENTIONS.117.005119 28302643

[6] Vanzetto G Berger-Coz E Barone-Rochette G, et al Prevalence, therapeutic management and medium-term prognosis of spontaneous coronary artery dissection: results from a database of 11,605 patients. Eur J Cardiothorac Surg. 2009;35:250-254. doi:10.1016/j.ejcts.2008.10.023 19046896

[7] Mortensen KH Thuesen L Kristensen IB Christiansen EH. Spontaneous coronary artery dissection: a Western Denmark Heart Registry study. Catheter Cardiovasc Interv. 2009;74:710-717. doi:10.1002/ccd.22115 19496145

[8] Faden MS Bottega N Benjamin A Brown RN. A nationwide evaluation of spontaneous coronary artery dissection in pregnancy and the puerperium. Heart. 2016;102:1974-1979. doi:10.1136/heartjnl-2016-309403 27411842

[9] Vijayaraghavan R Verma S Gupta N Saw J. Pregnancy-related spontaneous coronary artery dissection. Circulation. 2014;130:1915-1920. doi:10.1161/circulationaha.114.011422 25403597

[10] Saw J Sedlak T Aymong E, et al Spontaneous coronary artery dissection outcomes and association with fibromuscular dysplasia. Can J Cardiol. 2013;29:S256. doi:10.1016/j.cjca.2013.07.421

[11] Bonnet J Aumailley M Thomas D Grosgogeat Y Broustet JP Bricaud H. Spontaneous coronary artery dissection: case report and evidence for a defect in collagen metabolism. Eur Heart J. 1986;7:904-909. doi:10.1093/oxfordjournals.eurheartj.a061979 3792352

[12] Wingrove CS Garr E Godsland IF Stevenson JC. 17beta-oestradiol enhances release of matrix metalloproteinase-2 from human vascular smooth muscle cells. Biochim Biophys Acta. 1998;1406:169-174. doi:10.1016/s0925-4439(97)00097-5 9573355

[13] Cade JR Szarf G de Siqueira MEM, et al Pregnancy-associated spontaneous coronary artery dissection: insights from a case series of 13 patients. Eur Heart J Cardiovasc Imaging. 2017;18:54-61. doi:10.1093/ehjci/jew021 26928981

[14] Higgins GL 3rd Borofsky JS Irish CB Cochran TS Strout TD. Spontaneous peripartum coronary artery dissection presentation and outcome. J Am Board Fam Med. 2013;26:82-89. doi:10.3122/jabfm.2013.01.120019 23288285

[15] Sheikh A O′Sullivan M. Pregnancy-related spontaneous coronary artery dissection: two case reports and a comprehensive review of literature. Heart Views. 2012;13:53. doi:10.4103/1995-705x.99229 22919449 PMC3424780

[16] Koller PT Cliffe CM Ridley DJ. Immunosuppressive therapy for peripartum-type spontaneous coronary artery dissection: case report and review. Clin Cardiol. 1998;21:40-46. 9474465 10.1002/clc.4960210108 PMC6656028

[17] Ito H Taylor L Bowman M Fry ET Hermiller JB Van Tassel JW. Presentation and therapy of spontaneous coronary artery dissection and comparisons of postpartum versus nonpostpar tum cases. Am J Cardiol. 2011;107:1590-1596. doi:10.1016/j.amjcard.2011.01.043 21439531

[18] Havakuk O Goland S Mehra A Elkayam U. Pregnancy and the risk of spontaneous coronary artery dissection: an analysis of 120 contemporary cases. Circ Cardiovasc Interv. 2017;10:e004941. doi:10.1161/circinterventions.117.004941 28302642

[19] Elkayam U Havakuk O. Pregnancy-associated coronary artery dissection: a therapeutic dilemma. J Am Coll Cardiol. 2018;71:469-470. doi:10.1016/j.jacc.2017.09.1152 29389369

[20] Hassan S Prakash R Starovoytov A Saw J. Natural history of spontaneous coronary artery dissection with spontaneous angiographic healing. JACC Cardiovasc Interv. 2019;12:518-527. doi:10.1016/j.jcin.2018.12.011 30826233

[21] ACOG Practice Bulletin No. 202: Gestational Hypertension and Preeclampsia. Obstet Gynecol. 2019;133:e1-e25. doi:10.1097/AOG.0000000000003018 10.1097/AOG.0000000000003018 30575675

[22] Wheeler TL 2nd Blackhurst DW Dellinger EH Ramsey PS. Usage of spot urine protein to creatinine ratios in the evaluation of preeclampsia. Am J Obstet Gynecol. 2007;196:465.e1-e4. doi:10.1016/j.ajog.2006.10.892 17466704

[23] Verbruggen M Mannaerts D Muys J Jacquemyn Y. Use of ticagrelor in human pregnancy, the first experience. BMJ Case Rep. 2015;2015:bcr2015212217. doi:10.1136/bcr-2015-212217 PMC4680596 26607190

[24] Myers B Neal R Myers O Ruparelia M. Unplanned pregnancy on a direct oral anticoagulant (rivaroxaban): a warning. Obstet Med. 2016;9:40-42. doi:10.1177/1753495X15621814 27512489 PMC4950440

[25] Saw J Humphries K Aymong E, et al Spontaneous coronary artery dissection: clinical outcomes and risk of recurrence. J Am Coll Cardiol. 2017;70:1148-1158. doi:10.1016/j.jacc.2017.06.053 28838364

[26] De Santis M De Luca C Mappa I, et al Clopidogrel treatment during pregnancy: a case report and a review of literature. Intern Med. 2011;50:1769-1773. doi:10.2169/internalmedicine.50.5294 21841343

[27] Rowan JA McLintock C Taylor RS North RA. Prophylactic and therapeutic enoxaparin during pregnancy: indications, outcomes and monitoring. Aust N Z J Obstet Gynaecol. 2003;43:123-128. 14712967 10.1046/j.0004-8666.2003.00034.x

[28] Tello-Montoliu A Seecheran NA Angiolillo DJ. Successful pregnancy and delivery on prasugrel treatment: considerations for the use of dual antiplatelet therapy during pregnancy in clinical practice. J Thromb Thrombolysis. 2013;36:348-351. doi:10.1007/s11239-012-0830-7 23143651

